# CRISPR/Cas-Mediated Optimization of Soybean Shoot Architecture for Enhanced Yield

**DOI:** 10.3390/ijms26167925

**Published:** 2025-08-16

**Authors:** Nianao Li, Xi Yuan, Bei Han, Wei Guo, Haifeng Chen

**Affiliations:** 1 Key Laboratory of Biology and Genetic Improvement of Oil Crops, Ministry of Agriculture and Rural Affairs, Oil Crops Research Institute, Chinese Academy of Agricultural Sciences, Wuhan 430062, China; 82101235162@caas.cn (N.L.); yuanxi193@outlook.com (X.Y.); hanbei@caas.cn (B.H.); 2 Graduate School of Chinese Academy of Agricultural Sciences, Beijing 100081, China

**Keywords:** CRISPR/Cas, gene editing, soybean, plant architecture, yield optimization

## Abstract

Plant architecture is a crucial agronomic trait significantly impacting soybean (*Glycine max*) yield. Traditional breeding has made some progress in optimizing soybean architecture, but it is limited in precision and efficiency. The Clustered Regularly Interspaced Short Palindromic Repeats and CRISPR-associated protein (CRISPR/Cas) system, a revolutionary gene-editing technology, provides unprecedented opportunities for plant genetic improvement. This review outlines CRISPR’s development and applications in crop improvement, focusing specifically on progress regulating soybean architecture traits affecting yield, such as node number, internode length, branching, and leaf morphology. It also discusses the technical challenges for CRISPR technology in enhancing soybean architecture, including that the regulatory network of soybean plant architecture is complex and the development of multi-omics platforms helps gene mining. The application of CRISPR enables precise the regulation of gene expression through promoter editing. Meanwhile, it is also faced with technical challenges such as the editing of homologous genes caused by genome polyploidy, the efficiency of editing tools and off-target effects, and low transformation efficiency. New delivery systems such as virus-induced genome editing bring hope for solving some of these problems. The review emphasizes the great potential of CRISPR technology in breeding next-generation soybean varieties with optimized architecture to boost yield potential.

## 1. Introduction

Plant architecture has a significant impact on crop yield since it influences light interception, nutrient allocation, and adaptability to high-density planting. The Green Revolution is a typical case of plant architecture. It involved the introduction of semi-dwarf wheat (*Triticum aestivum*) and rice (*Oryza sativa*) varieties with optimized plant height and strong stems, which led to a huge increase in global grain production [1,2,3]. These architectural changes improved lodging resistance and resource utilization efficiency, resulting in higher yields under intensive farming conditions. Researchers have found that the microRNA319/GIBBERELLIN MYB (*miR319/TaGAMYB3*) module in wheat regulates traits like the number of tillers and the growth of spikes, which directly affects yield [4]. The Quantitative Trait Locus for Grain Length and Filling Factor on Chromosome 5 (*qGLF5*) gene from *Oryza rufipogon* makes rice kernels and plants better shaped, which increases productivity [5]. These examples show how important plant structure is for determining yield and show that genetic methods could be used to improve crop performance.

To further advance the optimization of plant architecture in crops, building on the success of architectural optimization in crops like wheat and rice, the Clustered Regularly Interspaced Short Palindromic Repeats (CRISPR) and CRISPR-associated protein (CRISPR/Cas) system has emerged as a revolutionary tool in genetic engineering, offering precise, versatile, and efficient methods for targeted genome editing. CRISPR/Cas-based knockout of Respiratory Burst Oxidase Homolog B (*OsRbohB*) in rice significantly improved plant heat stress tolerance and yield attributes such as spikelet filling and grain weight, illustrating the system’s capability to modulate both abiotic resilience and architecture-related traits [6]. Furthermore, the systematic editing of the Cytokinin Oxidase/Dehydrogenase (*OsCKX*) gene in rice family members has shown to fine-tune rice height, panicle size, and grain number, demonstrating direct architectural reshaping via CRISPR/Cas strategies [7]. These studies underscore how CRISPR/Cas technologies transcend trait-specific modifications, establishing themselves as powerful instruments for modern crop design.

Beyond traditional gene knockout approaches, CRISPR/Cas applications now include advanced techniques such as base editing, prime editing, and gene expression modulation, significantly expanding its utility in crop improvement. Recent studies demonstrate that these tools enable precise single-nucleotide modifications and transcriptional regulation without inducing DNA double-strand breaks (DSBs), thereby increasing editing safety and efficiency [8,9]. By integrating CRISPR/Cas with genomic selection and transformation systems, soybean varieties with optimized architecture and environmental resilience are being developed, addressing modern agricultural challenges. This review aims to provide an overview of the recent progress in utilizing CRISPR/Cas technology to precisely manipulate key genes involved in soybean architecture, highlighting both the achievements and the remaining challenges in this field.

## 2. Evolution of CRISPR/Cas Technology

### 2.1. From Prokaryotic Immunity to Programmable Nucleases

CRISPR was first noticed as unusual direct repeats in *Escherichia coli* [10] and later shown to colocalize with Cas genes [11]. Mojica and colleagues linked CRISPR spacers to invading phage DNA, proposing an RNA-mediated defense system [12]. This hypothesis was mechanistically confirmed when [13] demonstrated that the Cas9–CRISPR RNA (CrRNA)–trans-activating CRISPR RNA (tracrRNA) complex introduces DSBs at sites flanked by an NGG protospacer-adjacent motif (PAM). Engineering a single-guide RNA (sgRNA) of 20 nt streamlined the system for laboratory use and, within one year, Zhang and Church laboratories showed its functionality in mammalian cells [14,15], igniting a genome-editing revolution.

### 2.2. Diversifying the CRISPR Toolbox

**Figure 1:**
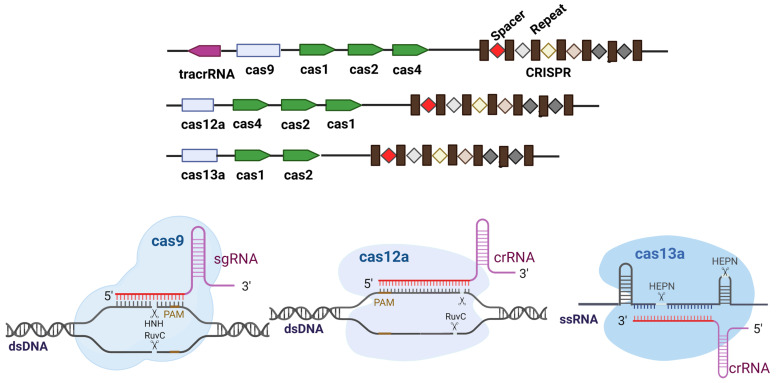
Genomic organization and functional mechanisms of representative Class 2 CRISPR–Cas systems.

**Figure 2:**
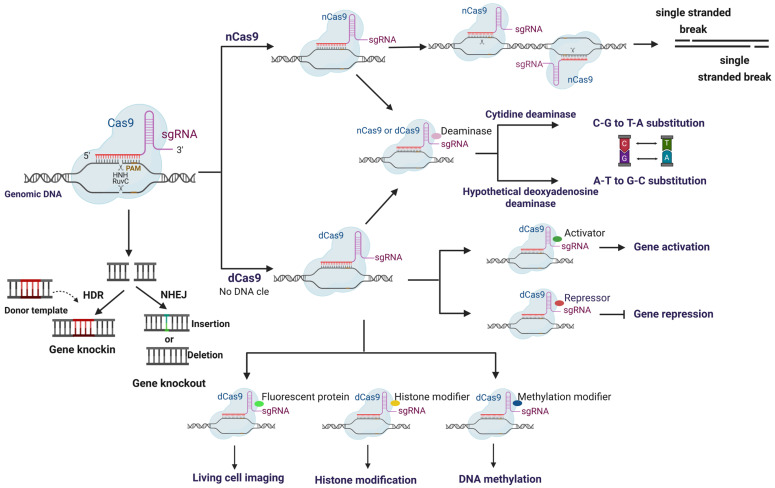
Functional mechanisms and diverse applications of wild-type Cas9, nickase variants, and dCas9 in CRISPR-based genome engineering.

Subsequent engineering has broadened the precision and scope of the CRISPR platform. CRISPR systems are broadly classified based on effector module architecture, with Class 1 systems (Types I, III, and IV) employing multi-subunit protein complexes and Class 2 systems (Types II, V, and VI) utilizing single effector proteins [16,17] (Figure 1). High-fidelity *Streptococcus pyogenes* Cas9 (SpCas9) variants, such as enhanced specificity Cas9 (eSpCas9) and hyper-accurate Cas9 (HypaCas9), carry structure-guided mutations that suppress off-target cleavage [18,19], addressing one of the primary concerns in precise genome editing applications. The recently developed SpCas9 with relaxed PAM recognition (SpRY) variant further expands the targetable genomic regions by relaxing PAM requirements, enabling plant genome editing at sites previously inaccessible to conventional SpCas9 [20]. Catalytically impaired derivatives, nickase Cas9 (nCas9) and nuclease-dead Cas9 (dCas9), enable homology-directed repair bias or programmable transcriptional/epigenetic regulation [21,22] (Figure 2). CRISPR/dCas9-based transcriptional regulation systems represent another frontier in the genetic modulation of crops, with activators and repressors enhancing gene activation and interference [23]. These systems are particularly valuable for modulating gene expression without permanently altering DNA sequences, allowing for more nuanced control of plant traits. Building upon these modifications, precision editors emerged by fusing deaminases or reverse transcriptase to nCas9, leading to cytosine/adenine base editing [24,25] and prime editing for small insertions, deletions, or substitutions [26].

The parallel discovery of Class 2 alternatives has significantly increased targeting flexibility beyond that of the traditional SpCas9 system. Cas12a (Cas12a formerly Cpf1) recognizes 5′-TTTV PAMs and generates staggered ends [27], offering advantages in T-rich genomic regions that are common in plant promoters and introns. The more recently characterized Cas12h is a compact DNA cutter for only one strand of double-stranded DNA, allowing for precise gene editing [28]. It has been successfully engineered into an ABE-Cas12h for efficient A-to-G conversion. Cas13 targets RNA rather than DNA [29], opening new possibilities for the transient modification of gene expression. New variants of the CRISPR system are continuously emerging to improve the efficiency and precision of gene editing and reduce potential risks, thereby broadening the application prospects of CRISPR technology (Table 1).

### 2.3. Adaptation to Plant Genomes

Applying CRISPR to plant genomes requires adaptations to address polyploidy, cell wall composition, and genotype-dependent transformation. Early successes in Arabidopsis (*Arabidopsis thaliana*) and rice have been extended to diverse crops, with pooled CRISPR-Cas9 approaches enabling multiplex mutagenesis for large-scale functional genomic studies [56,57]. Compact nucleases like Cas12f derived from Acidibacillus sulfuroxidans, one of the smallest Cas proteins, can be maintained in Potato Virus X (PVX) vectors for plant genome editing [58]. Additionally, “CRISPR-Combo” systems integrate multiple editing capabilities, expanding the plant genome-editing toolbox [59]. While these innovations show remarkable potential, their agricultural applications hinge on overcoming delivery barriers through advanced transformation engineering.

Recent advancements in genetic manipulation techniques are closely linked to progress in transformation engineering methods. For instance, *BBM* and Wuschel (*WUS*) have significantly improved transformation efficiency in crops, such as maize (*Zea mays*), rice, and wheat [60]. In addition, the Wuschel-Related Homeobox 5 (*TaWOX5*) gene in wheat has been identified as an enhancer of transformation and regeneration [61]. To overcome the limitations of traditional transformation methods, novel strategies have been developed, including Ribonucleoprotein based CRISPR/Cas delivery systems and geminivirus replicons for the transient amplification of donor templates. These approaches have demonstrated high efficiency in crops [62,63,64,65]. Tissue culture-free genetic modifications have been achieved through virus-induced genome editing, grafting, and cut-dip-budding techniques [63,66]. Although transformation efficiency varies significantly among crops, the ongoing optimization of nuclease engineering, multiplex design, and innovative delivery methods is enhancing the toolkit for crop genetic improvement, unlocking the full potential of CRISPR technology in agriculture.

## 3. Applications of CRISPR/Cas in Crop Improvement

Since its initial application in wheat for powdery mildew resistance [67], CRISPR/Cas has surpassed zinc-finger nucleases and Transcription Activator-Like Effector Nucleases (TALENs) to become the cornerstone of genome editing [68]. A single sgRNA directs SpCas9 to its target with precision [69], streamlining edits that once demanded complex protein engineering [70]. Its multiplexing ability, targeting six to eight sites in a single construct [70], combined with per-target costs below USD 300 [71], has significantly shortened breeding cycles. For instance, the Mildew Resistance Locus O (MLO) knockout in wheat reduced resistance breeding from twelve years to just two [70]. Despite the high efficiency and accuracy of CRISPR/Cas9, the precision of editing and the occurrence of off-target effects can vary based on the target site and the design of the sgRNA [71,72].

CRISPR’s versatility shines across crops like fruits, cereals, and oilseeds. In tomato (*Solanum lycopersicum*), de novo domestication of wild varieties via CRISPR/Cas9 enhanced fruit size, yield, and nutrition by targeting multiple yield-related genes [73,74]. In wheat, editing all three homoeologs of CBL interacting protein kinases 14 (*TaCIPK14*) conferred broad-spectrum stripe-rust resistance [75]. These examples underscore the power of CRISPR to improve stress resistance, productivity, and quality. Recent advances enable precise control of quantitative traits. Prime editing in rice, inserting AAA into the 5′UTR of silicone breast implants (*OsSBI*) and TTT into High Tillering and Dwarf 1 (*OsHTD1*), fine-tuned translation efficiency to achieve semi-dwarf, high-tillering Green Revolution traits [76]. This shift from knockouts to promoter-level optimization broadens CRISPR’s breeding potential. Beyond DNA edits, CRISPR/dCas9 focuses on gene expression regulation. In tomato, CRISPR/dCas12a with epigenetic modifiers activated Phenylalanine Ammonialyase 2 (*SlPAL2*), enhancing bacterial wilt resistance without yield penalties [77,78]. In poplar (*Populus*), CRISPRa activated Targeting Protein for Xklp2 (*TPX2*) and a G-type lectin receptor-like protein kinase (*LecRLK-G*), boosting growth and stress resistance [79]. This reversible, cleavage-free approach to transcriptional control offers vast potential for crop improvement.

The transformative impact of CRISPR on crop improvement, from precise trait enhancement to innovative gene regulation, continues to redefine agricultural breeding with unparalleled efficiency and versatility. The following content focuses on the application of CRISPR/Cas in altering the plant architectural traits of soybeans.

## 4. Broad Applications of CRISPR/Cas in Soybean Improvement

The application of CRISPR/Cas9 to soybean began in 2015 with the successful targeted mutagenesis of a Green Fluorescent Protein (GFP) gene [80]. This milestone was rapidly followed by validation studies using hairy root, protoplast, and stable transformation systems, which confirmed the technology’s feasibility and reliability in soybean [81,82]. Since these foundational demonstrations, CRISPR/Cas technologies have been swiftly deployed to enhance a wide spectrum of crucial agronomic traits (Table 2).

A primary focus has been fortifying soybean resilience against environmental stressors. For abiotic stress, editing key regulatory genes like Homeodomain-leucine Zipper 4 (*GmHdz4*), Abscisic Acid-Induced Transcription Repressors (*GmAITR*), and Conglycinin (*GmCG*) has significantly improved tolerance to drought and salinity in soybean [83,84,85]. In parallel, biotic stress resistance has been achieved through strategies such as engineering durable resistance to powdery mildew by modifying *MLO* homologs [86] and enhancing defenses against the soybean mosaic virus via the mutagenesis of flavonoid biosynthesis genes like Flavanone 3-Hydroxylase 1 *(GmF3H1*), *GmF3H2*, and Flavone Synthase II-1 (*GmFNSII-1*) [87]. More recently, targeted disruption of UDP-Glucosyltransferase (*GmUGT*) was shown to bolster resistance to leaf-chewing insects by modulating flavonoid pathways [88].

Improving seed quality and nutritional value has been another key objective. Notable achievements include the development of high-oleic acid soybeans by editing Fatty Acid Desaturase (*GmFAD2*) homologs [89] and the removal of the characteristic beany flavor through knockout of Lipoxygenase (*Lox*) genes [90]. Furthermore, the nutritional profile has been enhanced by reducing anti-nutritional compounds like raffinose family oligosaccharides [91]. A pioneering strategy recently integrated AlphaFold protein structure predictions with CRISPR/Cas9 to engineer novel alleles of *Sugars* Will Eventually be Exported Transporter (*GmSWEET10*) genes. This approach successfully increased seed oil content in the field without a yield penalty, demonstrating a powerful new paradigm for customized gene design that transcends the limits of traditional breeding [92].

Beyond defense and quality, CRISPR has been instrumental in engineering complex yield-related traits. Direct yield components, such as grain size and weight, have been successfully increased by mutating Enhancer of Organ Size 1 (*GmEOD1*), a negative regulator of seed development [93]. Addressing a more systemic challenge, researchers have resolved the long-standing trade-off between high yield and high protein content. By editing Clavata3/Embryo Surrounding Region-related (*RIC*) genes to optimize symbiotic nodulation and carbon-nitrogen balance, they achieved simultaneous improvements in both yield and protein levels in field trials [94].

These collective advancements, spanning from stress resilience to end-product quality and complex yield physiology, underscore the precision and versatility of CRISPR/Cas in soybean breeding. Building on this robust foundation, the deliberate engineering of shoot architecture now stands out as a pivotal frontier for maximizing soybean’s yield potential.

**Table 2:** Summary of recent applications of CRISPR/Cas-mediated gene editing for soybean trait improvement (2020–2025).

Trait Category	Target Gene(s)	Modification	Key Phenotypic Outcome(s)	Reference
**Abiotic Stress Resistance**				
Drought resistance	*GmHdz4*	Knockout	Enhanced drought tolerance	Zhong et al. [83]
Salt tolerance	*GmAITR2, GmAITR3, GmAITR4, GmAITR5, GmAITR6*	Multiplex knockout	Enhanced salt tolerance (germination, seedling, and field)	Wang et al. [84]
Salt tolerance/quality improvement	*GmCG-1, GmCG-2, GmCG-3*	Knockdown	Reduced β-conglycinin; increased protein and sulfur-amino acids; enhanced salt tolerance (germination/seedling)	Yang et al. [85]
Multiple stress tolerance	*GmARM*	Knockout	Enhanced tolerance to salt, alkali, and pathogens	Luo et al. [95]
**Biotic Stress Resistance**				
Soybean mosaic virus resistance	*GmF3H1* *GmF3H2* *GmFNSII-1*	Multiplex knockout	Enhanced resistance to soybean mosaic virus	Zhang et al. [87]
Powdery mildew resistance	*GmMLO02* *GmMLO19* *GmMLO20* *GmMLO23*	Multiplex knockout	Enhanced powdery mildew resistance	Bui et al. [86]
Chewing insects resistance	*GmUGT*	Knockout	Enhanced resistance to cotton bollworm and armyworm	Zhang et al. [88]
Root rot disease resistance	*GmTAP1*	Knockout	Enhanced resistance to multiple *Phytophthora sojae* biotypes	Liu et al. [96]
Soybean cyst nematode resistance	*GmSNAP02*	Knockout	Enhanced resistance to cyst nematodes	Usovsky et al. [97]
Insect resistance/flowering time regulation	*GmCDPK38*	Knockout	Delayed flowering and enhanced resistance to Spodoptera litura	Li et al. [98]
**Soybean Quality Improvement**				
Increased Oil Content	*GmSWEET10a, GmSWEET10b*	Gene editing (informed by AlphaFold)	Increased oil content	Wang et al. [92]
	*GmSFAR4a, GmSFAR4b*	Knockout	Increased oil content	Liao et al. [99]
Removal of beany flavor	*GmLox1, GmLox2, GmLox3*	Multiplex knockout	Elimination of beany flavor	Wang et al. [90]
Reduced soy allergenicity	*GmP34, GmP34h1, GmP34h2*	Multiplex knockout	Reduced *GmP34* allergen potential	Baek et al. [100]
Reduce anti-nutritional factors	*GmRS2* *GmRS3*	Multiplex knockout	Reduced raffinose family oligosaccharides	Cao et al. [91]
**Other**				
Pod shattering resistance	*GmPDH1*	Knockout	Increased pod-shattering resistance	Zhang et al. [101]
Increased seed size	*GmEOD1*	Knockout	Increased seed size and hundred-seed weight	Yu, et al. [93]
Herbicide tolerance	*GmALS1, GmALS3*	Base editing (C-to-T)	Increased herbicide resistance without yield penalty	Niu et al. [102]
Enhanced symbiotic nitrogen fixation	*GmRIC1*, *GmRIC2*	Knockout	Increased nodule number; balanced C/N allocation	Zhong, et al. [94]

Abbreviations: *GmHdz4—Glycine max* Homeodomain-leucine Zipper 4; *GmARM—Glycine max* Armadillo Repeat Motif-containing gene; *GmTAP1—Glycine max* Transcriptionally Active Protein 1; *GmSNAP02—Glycine max* Soluble NSF Attachment Protein 02; *GmCDPK38—Glycine max* Calcium-Dependent Protein Kinase 38; *GmSFAR4a—Glycine max* Seed Fatty Acid Reducer 4a; *GmP34—Glycine max* P34; *GmRS2*--*Glycine max* Raffinose Synthase 2; *GmPDH1—Glycine max* Prephenate Dehydrogenase 1; *GmALS1*: *Glycine max* Acetolactate Synthase 1.

## 5. CRISPR/Cas Applications in Soybean Shoot Architectural Improvement

**Figure 3:**
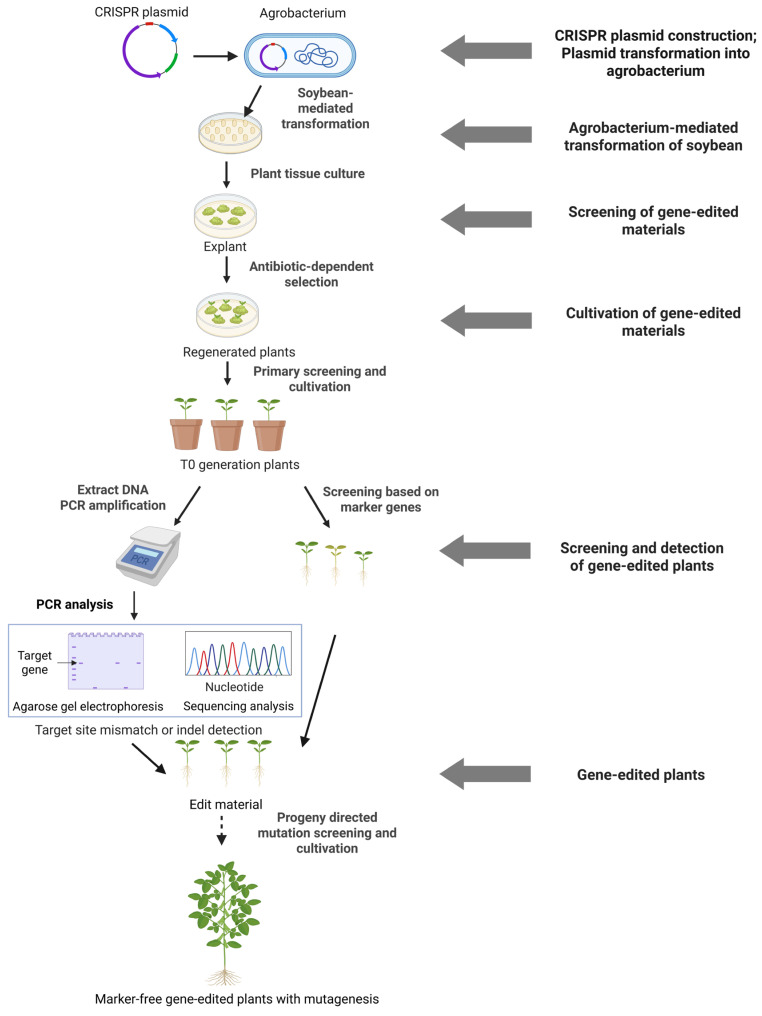
Workflow of CRISPR–Cas-mediated gene editing in soybean using Agrobacterium transformation.

The Green Revolution has demonstrated that optimizing plant architecture is an effective way to increase crop yields [103]. However, soybeans possess architectural characteristics that differ from those of gramineous crops such as rice and wheat. In soybean, pods are borne on the nodes, and the yield-associated architectural traits primarily include six interrelated components: plant height, number of nodes, stem growth habit (indeterminate, semi-determinate, or determinate), leaf morphology and petiole angle, and number of seeds per pod [104,105]. With the development of CRISPR/Cas systems, precise editing of single genes controlling these traits has become feasible, enabling the rapid assembly of desirable phenotypes (Figure 3).

### 5.1. Precision Engineering of Node Number

Node number in soybean—a critical determinant of yield—is regulated by a complex network of developmental pathways involving floral transition, vegetative growth duration, and stem architecture [106]. Advances in CRISPR/Cas9 genome editing have enabled precise manipulation of these pathways, offering new strategies for optimizing plant structure and productivity.

Central to this regulation are florigen genes, particularly Flowering Locus T (FT) homologs. The FT protein, and in some cases, its mRNA, functions as mobile flowering signals that primarily transported from leaves to the shoot apical meristem to trigger the reproductive transition [107]. In soybean, GmFT2a acts as a classical photoperiod-sensitive florigen primarily promoting flowering under short-day conditions by interacting with transcription factors such as Floral Development Locus 19 (GmFDL19) to activate downstream floral integrator genes including Apetala1 (GmAP1) and Suppressor of Overexpression of Constans 1 (GmSOC1) [108,109,110]. CRISPR/Cas9-mediated knockout of GmFT2a delayed flowering, extending the vegetative phase and increasing node number [109,110,111]. GmFT5a functions as both a floral promoter and determinacy regulator, modulating shoot architecture by disrupting the Dt1–AP1 feedback loop [112]. Loss-of-function GmFT5a mutations delay flowering and can disrupt post-flowering stem termination, leading to a shift from determinate to indeterminate growth habits, which enables continued node production beyond the reproductive phase, particularly under specific photoperiod conditions [109,110]. The sequential activation of these florigen genes creates a developmental window during which node formation continues, directly linking flowering gene expression kinetics to final node number.

In parallel, the circadian clock machinery contributes to architectural regulation through its influence on growth patterns. Mutations in Late Elongated Hypocotyl (*GmLHY*), a key circadian regulator, resulted in reduced plant height and shortened internodes, likely via disruption of gibberellin (GA) signaling pathways [113]. Direct manipulation of transcription factors involved in developmental transitions has also produced substantial architectural changes. Targeted editing of *AP1* homologs generated quadruple mutants that displayed delayed flowering under short-day conditions, increased plant height, and resulted in a greater internode length and a higher number of nodes [114]. Similarly, CRISPR/Cas9-mediated mutagenesis of the Squamosa Promoter Binding Protein-like 9 (*GmSPL9*) gene family (*GmSPL9a–d*) enhanced branching and increased node numbers, with each homolog contributing differentially to overall plant architecture [115].

Collectively, these studies highlight how integrated control of flowering, stem growth, and circadian regulation underlies node number variation in soybean. The precision of CRISPR/Cas9 technology in targeting these interconnected pathways presents a powerful approach for developing soybean cultivars with improved yield potential and adaptability.

### 5.2. Precision Engineering of Internode Length

Optimizing soybean plant architecture requires precision engineering of internode length, as this directly affects plant height and lodging resistance. The GA pathway serves a central regulatory function in plant height, with its biosynthesis, metabolism, and signaling pathways being critical determinants of stem elongation [116,117]. Loss-of-function mutations in Dwarf1 (*GmDW1*), identified from EMS-induced mutants, result in dwarf phenotypes in soybean by disrupting GA biosynthesis [118]. Advances in CRISPR/Cas technology have facilitated the targeted modification of key GA-related genes to achieve desired plant heights. For instance, CRISPR-mediated mutagenesis of *GmLHY* genes, which encode myeloblastosis (MYB) transcription factors, significantly reduces plant height by modulating the GA pathway in soybean [113]. Additionally, *AP1* homologs in soybean, such as *GmAP1s*, influence internode length through GA biosynthesis; a quadruple *gmap1* mutant showed longer internodes and increased plant height, while overexpression of *GmAP1a* led to shorter internodes [114]. Conversely, targeting GA catabolism genes provides an alternative method for height control. Studies on Gibberellin 2-oxidase 8 (*GA2ox8A* and *GA2ox8B*), which encode GA2-oxidases involved in degrading active GAs, indicate that higher copy numbers of these genes are associated with reduced shoot length, a trait selected during soybean domestication [119].

Light conditions, particularly in dense plantings, trigger shade-avoidance responses that can result in undesirable internode elongation. Blue light photoreceptors, such as Cryptochrome 1s (*GmCRY1s*) in soybean, play a crucial role in repressing excessive stem elongation under low blue light conditions by stabilizing the basic Leucine Zipper (bZIP) transcription factors TGACG-motif-binding Factor 1 and 2 (STF1 and STF2), which, in turn, upregulate *GA2ox* expression and reduce the levels of bioactive GAs [120]. The photomorphogenic repressor Constitutive Photomorphogenic 1b (*GmCOP1b*) promotes stem elongation under light conditions; CRISPR-created *gmcop1b* mutants exhibited reduced shade responses and improved performance in high-density planting [121]. The Plant Height 13 (*PH13*) gene and its paralog *PHP* (*PH13* Paralog Homolog) are also key regulators. PH13 affects internode length by interacting with GmCOP1 proteins to modulate the accumulation of STF1/STF2, which are vital for GA metabolism [122,123]. Knocking out *PH13* and *PHP* using CRISPR/Cas9 resulted in optimized internode lengths, enhancing lodging resistance and increasing yields in dense cultivation [124]. Furthermore, the reduced internode 1 (*rin1*) gene, which encodes a SUPPRESSOR OF PHYA 105 3a (SPA3a) protein, impacts internode length by regulating *GA2ox7* expression via STF1/STF2, linking light signaling components to GA-mediated growth control [123].

Additional pathways and environmental responses also regulate internode length. The brassinosteroid (BR) pathway is critical for cell elongation and division. For example, CRISPR/Cas9-mediated editing of DWARF1(*GmDWF1*) genes (*GmDWF1a* and *GmDWF1b*), which encode key enzymes in BR biosynthesis, has successfully induced dwarfism in soybean, highlighting the potential for BR pathway manipulation to create compact plant architectures suitable for high-density planting [125]. Moreover, plant responses to Ultraviolet-B (UV-B) radiation can influence plant height. In soybean, the *E3* ubiquitin ligase Increased Leaf Petiole Angle 1 (*GmILPA1*) is upregulated under UV-B exposure, promoting the ubiquitination and degradation of the GA catabolic enzyme GmGA2ox-like. This action counters the UV-B-induced reduction in bioactive GAs, thus promoting plant height. Conversely, *gmilpa1* mutants exhibit dwarfism under UV-B conditions, underscoring *GmILPA1*’s role in maintaining GA homeostasis and growth under UV-B stress [126]. These findings illustrate the diverse molecular targets available for fine-tuning soybean internode length and overall plant height through precision gene editing.

In the protein degradation pathway, *PH13* and its paralog *PHP* function as key regulators of soybean branching architecture. *PH13* encodes a WD40-repeat domain protein homologous to Arabidopsis Suppressor of Phya-105 (SPA) family proteins. Additional pathways and environmental responses also regulate internode length. The *GmAP1* genes act as negative regulators of branch formation, as quadruple *gmap1* mutants exhibit significantly increased branch numbers, whereas *GmAP1a*-overexpressing lines show reduced branching [127].

### 5.3. Precision Editing of Branching Patterns

Branching architecture fundamentally determines soybean canopy structure, light interception efficiency, and yield potential [128]. CRISPR/Cas technology has emerged as a powerful tool for the precise modification of genes that control branch development.

Several key regulatory genes have been identified as targets for optimizing branch patterns. The *SPL9* gene family functions as a negative regulator of branch formation in soybean. While single mutants showed limited phenotypic changes, higher-order mutants exhibited significantly increased node and branch numbers [115]. The *Dt2–GmAP1* regulatory module represents a key mechanism controlling shoot branching in soybean. Through a genome-wide association study of 2409 soybean accessions, *Dt2* was identified as a major locus on chromosome 18 associated with branch number [127]. Functionally, *Dt2* interacts with Agamous-like 22 (*GmAGL22*) and *GmSOC1a* to form a transcriptional complex that directly binds to the promoters of *GmAP1a* and *GmAP1d*, activating their expression.

It forms an *E3* ubiquitin ligase complex with GmCOP1 proteins to target specific transcription factors, such as STF1/2, for degradation, thereby modulating plant architecture. The *phd* (*PH13*/*PHP* double mutant) plants exhibited multiple advantageous traits under high-density planting conditions, including increased branch number, more pods per plant, thicker stems, and a 15.8% improvement in grain yield per plant compared to the wild-type cultivar JY202 [124].

Hormone transport and signaling also play crucial roles in branch development. Pinformed 1(*GmPIN1*) genes encode auxin efflux carriers that control hormone distribution patterns in soybean. Zhang et al. found that *gmpin1abc* and *gmpin1bc* multiple mutants displayed more branches than wild-type plants [129]. In addition, these mutants showed shorter shoot stature and, in some lines, higher podding rates, indicating that *GmPIN1* regulates both branching and broader aspects of plant architecture.

The capacity of CRISPR/Cas9 technology to simultaneously target multiple genes represents a significant advantage in soybean enhancement. This multiplexing capability is essential for addressing genetic redundancy within the complex soybean genome, where the modification of a single gene often leads to minimal phenotypic changes due to the compensatory functions of homologous genes.

### 5.4. Precision Editing of Leaf Structure and Petiole Angle

Optimizing leaf orientation and petiole inclination is crucial for maximizing light interception and photosynthetic efficiency in soybean canopies [130]. However, conventional breeding efforts are often constrained by pleiotropic loci that impose trade-offs between leaf morphology and yield [131]. The advent of CRISPR/Cas9 genome editing offers new avenues to target these multifunctional genes with high precision, potentially circumventing such limitations.

A paradigmatic example is the *JAGGED1* (*Ln*) locus, which encodes a transcription factor that simultaneously regulates leaflet width and the number of seeds per pod (NSPP). The dominant Ln allele confers broad leaflets but reduces NSPP, whereas the recessive ln allele results in narrow leaflets accompanied by increased seed set employed CRISPR/Cas9 in the low-latitude soybean variety ‘Huachun 6′ to knock out *GmJAG1* using dual sgRNAs targeting two exons [132,133]. The resultant *gmjag* lines displayed the characteristic narrow-leaflet morphology and achieved an 8.7–8.8% yield increase under field conditions. While this work confirms the pleiotropic role of *GmJAG1*, it also illustrates that leaflet morphology and seed number remain coupled in the mutant lines, indicating that further allelic refinement or regulatory-level editing will be necessary to fully decouple these traits [133].

Beyond transcriptional regulators, hormone transport pathways also provide promising targets for architectural optimization. Zhang et al. demonstrated that multiplex CRISPR/Cas9 mutagenesis of the *GmPIN1* auxin efflux carriers disrupted polar auxin distribution at the petiole base, leading to more erect leaf angles favorable for high-density planting [129]. This work underscores the utility of targeting gene families with redundant functions in polyploid genomes to sculpt plant architecture.

Similarly, *GmILPA1* plays a critical role in modulating leaf petiole angle through control of pulvinus cell proliferation. A gamma ray-induced deletion in the fourth exon of *GmILPA1* was shown to increase petiole angles, and natural variation in its expression is inversely correlated with angle magnitude across diverse soybean accessions [134]. These findings point to *GmILPA1* as a promising candidate for promoter or base editing to achieve graded phenotypic outputs without altering protein coding sequences.

Collectively, these case studies highlight the transformative potential of CRISPR/Cas-based strategies in soybean canopy engineering. Representative target genes involved in regulating plant architecture and yield potential are summarized in Table 3. Through targeted gene disruption, multiplex editing, and regulatory sequence manipulation, researchers are now equipped to rationally redesign canopy traits to enhance light capture and yield efficiency in intensive cropping systems.

## 6. Challenges and Future Directions

### 6.1. Future Directions of CRISPR in Soybean Plant Architecture Regulation

The regulatory network of soybean plant architecture is intricate, with key genes often showing pleiotropic effects. For instance, the transcription factor *GmMYB14* influences leaf angle and enhances drought resistance by regulating stress-responsive pathways [141], while the soybean *Dt1* gene interacts with Flowering Locus D-class 1 (*FDc1*), *FT5a*, and *AP1* to form a complex network that governs reproductive transition and shoot apical meristem activity [112]. Chromatin topology and long-range chromosomal interactions also play roles in regulating gene expression during soybean polyploidization, diploidization, and domestication [119]. This complexity has spurred multi-omics advances; for instance, Li et al. developed an integrated database combining the deep sequencing of 984 germplasms, 2500+ phenotypic images, and existing multi-omics data to enable trait gene mining [142]. Concurrently, a cellular atlas of soybean was constructed using single-nucleus RNA sequencing, encompassing approximately 120,000 nuclei from 10 distinct organs and morphological structures [143], and another study mapped organ development via RNA sequencing [144]. Currently, multiple soybean multi-omics platforms have been developed to support soybean genetic research and breeding work. The main platforms established include SoyOmics, SoyFGB, SoyOD, SoyMD, etc. Integrating multidimensional omics data with artificial intelligence enables the identification of high-confidence target genes for CRISPR-based editing by reducing false positives, decoding complex regulatory landscapes, and facilitating precise functional validation. This approach supports precision breeding by prioritizing candidate genes across diverse biological layers [145].

The spatiotemporal regulation of plant growth genes is essential for organ development and environmental adaptation, with promoter regions critically orchestrating the timing and location of gene expression. For example, the Small Heat Shock Proteins (*SmsHSP24.1*) promoter in eggplant (*Solanum melongena*) harbors cis-regulatory elements that govern its spatiotemporal expression. A 2 kb promoter fragment was shown to drive heat-inducible expression in sensitive tissues, such as anthers and young leaves, while truncation analysis revealed a 300 bp core region responsible for basal activity and a distal 1.2 kb segment containing heat-responsive elements, illustrating promoter-mediated integration of environmental cues with tissue-specific expression [146]. Building on this understanding, CRISPR/Cas systems have enabled a range of promoter engineering strategies, from precise modifications of existing elements to the rational design of novel regulatory circuits.

A primary strategy for promoter engineering involves the targeted modification or disruption of existing cis-regulatory elements to precisely adjust a gene’s native expression pattern. This approach effectively fine-tunes transcriptional output. For instance, CRISPR/Cas9-mediated edits to the Nuclear Factor Y subunit C4 *(NF-YC4)* promoter in rice and soybean enhanced protein accumulation, leading to improved yield [147]. Similarly, precise editing of cis-regulatory elements within the promoter of the heat-inducible Casein Kinase I *(GhCKI)* gene in cotton (*Gossypium hirsutum*), using both CRISPR/Cas9 and CRISPR/Cpf1, disrupted MYB transcription factor binding sites. This modification attenuated high-temperature-induced *GhCKI* activation, thereby preserving male fertility under thermal stress [148].

A more advanced strategy focuses on designing and assembling synthetic regulatory circuits. This approach treats known cis-regulatory elements as modular units, combining them to create predictable and tunable expression patterns. Such modular design enables de novo programming of gene expression. A notable example is the use of prime editing, a highly precise CRISPR-based technique, to insert a carefully designed 10 bp heat-shock element into the targeted promoter region of the endogenous cell-wall invertase gene Cell Wall Invertase 5 (*LIN5*) in tomato, as well as in rice. This precise insertion rewired the native promoter, conferring heat-inducible expression that significantly enhanced yield resilience under thermal stress [149]. This work exemplifies the power of CRISPR technologies in enabling tailored genetic modifications and highlights the synthetic biology principle of modular design, offering a blueprint for engineering climate-resilient soybean cultivars.

The expanding toolkit of promoter engineering—from targeted edits of single elements to the modular construction of novel regulatory functions—provides unprecedented control over plant trait programming. Enabled by continuous advancements in CRISPR technologies, these strategies are pivotal for developing high-performing, resilient soybean cultivars capable of addressing future agricultural challenges.

### 6.2. Technical Challenges of CRISPR in Soybean

Soybean genome editing faces challenges due to its paleopolyploid genome, with comparative analyses indicating that approximately 75% of soybean genes exist in multiple copies [150]. On the one hand, this genetic redundancy necessitates simultaneous editing of homologous genes to observe phenotypic changes, as demonstrated in studies on *GmLHY* and *GmSPL9* genes. Quadruple mutants of *GmLHY* genes showed reduced plant height and shortened internodes [113], whereas higher-order mutants of *GmSPL9* genes exhibited altered plant architecture with increased node and branch numbers [115]. However, functional differentiation of homologous genes complicates selective editing [151,152]. The editing efficiency of CRISPR/Cas9 systems is constrained by both the GC content in regions proximal and distal to the PAM sequence and the preference for NGG PAM motifs, which together impose significant limitations on target-site selection [153]. The development of new editing tools has overcome the limitations of editing site selection. Next-generation tools like Cas12a and PAM-less SpRY offer expanded targeting capabilities [154,155] but introduce new challenges, such as potential off-target effects [156]. Additionally, although the study by Chen et al. has shown that the editing efficiency of the CRISPR-SpRY system in soybeans can reach up to 57.7%, there is still significant room for improvement in multiple aspects. First, target dependency is evident, with editing efficiency varying significantly among targets. Second, base editing efficiency remains low: A-to-G editing is only 5.0–5.6%, and C-to-T editing is mostly around 10%. Further, multi-gene editing efficiency is limited: While single-target editing is efficient, editing 4–6 genes simultaneously reduces biallelic mutation rates to 4–17% [156]. Further refinement is required for precise and efficient genome engineering of soybean.

The application of CRISPR-Cas in soybeans has long been hindered by transformation inefficiencies and genotype-dependent regeneration challenges [157]. Developmental genes such as GROWTH-REGULATING FACTOR5 have shown promise in enhancing soybean transformation [158,159]. This approach remains limited by chimerism that affects inheritance and the potential pleiotropic effects of GRF overexpression on plant development [159]. These limitations have spurred the exploration of alternative delivery systems such as virus-induced genome editing (VIGE).

VIGE has emerged as a promising strategy to overcome the need for stable transgene integration and to reduce regulatory barriers to generate gene-edited DNA-free plants [160,161,162]. In soybean, the Apple Latent Spherical Virus (ALSV) has been adopted as the first viral vector for gRNA delivery, successfully editing endogenous genes such as Phytoene desaturase [163]. VIGE systems demonstrate competitive editing efficiencies: In Solanaceous plants, Tobacco Rattle Virus (TRV)-based systems achieve 40.3% and PVX systems reach 36.5%, while the ALSV-based CCAC (Cas9-based Csy4-processed ALSV Carry) system shows 45.3% editing efficiency specifically in soybean hairy roots [107,163]. Beyond numerical efficiency, VIGE offers significant temporal advantages by eliminating the tissue culture period required for antibiotic selection in conventional Agrobacterium-mediated approaches. The CCAC system enables efficient gRNA delivery once a stable Cas9-expressing line is established, offering significant advantages for crops with low transformation rates like soybeans [163].

However, VIGE application in soybeans remains in its infancy, facing critical challenges, such as the efficient delivery of large Cas9 proteins and optimization of viral vectors for stable editing. The size constraints of viral cargo—Cas9 coding sequence (~4.2 kb) often exceeds viral packaging limits—pose significant hurdles, as does the requirement for transient expression without permanent genomic integration. The CCAC system addresses these through a hybrid approach: Cas9 is delivered via traditional Agrobacterium transformation while gRNAs are delivered through ALSV infection, utilizing the Csy4 endonuclease for precise gRNA release [163]. Additional strategies include engineering compact Cas variants (e.g., CasPhi), implementing split-Cas systems for reconstitution in planta, utilizing dual-virus approaches, and optimizing viral vector design through capsid engineering [164,165].

## 7. Conclusions

CRISPR-Cas technology represents a groundbreaking advancement in the field of agricultural biotechnology, offering significant potential for the improvement of soybean plant architecture. By enabling precise genetic modifications, this tool enhances vital agronomic traits, promoting the increased yield and adaptability of soybean crops. The application of CRISPR has allowed researchers to target key genes demonstrating the capability of this technology to refine plant structure and function effectively.

A critical consideration moving forward is how these CRISPR-mediated architectural improvements translate into tangible agronomic gains regarding yield, seed quality, and overall economic viability. While optimized architectures for high-density planting or lodging resistance are intrinsically linked to higher yield potential, the pleiotropic nature of many key architectural genes presents a notable challenge. This can create unintended trade-offs, for instance, between reduced plant height and final seed size. Herein lies a distinct advantage of precision genome editing. Unlike conventional breeding, which is often hampered by linkage drag, CRISPR/Cas systems enable the targeted introduction of favorable alleles or the fine-tuning of expression via promoter editing. These precise modifications offer a powerful means to uncouple desired architectural traits from negative pleiotropic consequences, paving the way for the co-optimization of plant form, yield, and quality in elite soybean cultivars.

Nevertheless, the implementation of CRISPR-Cas in soybeans is not without challenges. Issues such as off-target effects, the complexity of multi-gene editing, efficient delivery methods, and societal and regulatory acceptance present hurdles that must be addressed through ongoing research and development. The emergence of new Cas variants and advanced editing techniques continues to promise greater precision and efficiency in these endeavors. In conclusion, while hurdles persist, the progress being made in CRISPR-Cas technology offers a promising future for soybean development.

## Figures and Tables

**Table 1:** Advantages, disadvantages, and applications of CRISPR tools.

Tool	Enzyme(s)	Guide RNA	Target Type	PAM or PFS Site	Advantages	Disadvantages	Application Examples
CRISPR–Cas9	Cas9	sgRNA	dsDNA	PAM: typically 5′-NGG-3′ (SpCas9); varies by Cas9 variant	Simple design; high efficiency; multiplex capability for editing multiple targets; broad applicability [13]	Restricted by PAM; potentially high on-/off-target effects in some organisms [30,31]	Enhancing broad-spectrum resistance in rice varieties Kitaake, IR64, and Ciherang-Sub1 [32]; adjusting *TaGW2* dosage to increase wheat grain weight [33]
CRISPR–Cas12a	Cas12a (Cpf1)	crRNA	dsDNA/ssDNA	PAM: 5′-(T)TTN-3′	No need for tracrRNA; lower off-target rate; distinct recognition site from Cas9 [34,35]	Dependent on host DNA repair mechanisms; frequently results in small deletions (<100 bp) [36,37]	Site-directed mutagenesis of *OsDL* and *OsALS* genes in rice [38]
CRISPR-Cas12b	Cas12b (C2c1)	crRNA + tracrRNA or sgRNA	dsDNA/ssDNA	PAM: 5′-TTN-3′(species-dependent)	Higer temperature tolerance; compact size; high targeting specificity [39]	Less characterized; may require elevated temperatures for optimal activity	Enhancing broad-spectrum resistance in rice using AaCas12b-mediated editing of *OsEPFL9* and *OsGS3* [39]
CRISPR–Cas13	Cas13a/Cas13b (C2c2)	crRNA	ssRNA	PFS: 3′ non-G (bacteria); N/A in eukaryotes	PAM-independent; cleaves RNA only; high editing efficiency [29]	Potential nonspecific cleavage of bystander RNAs (collateral effect) [40]	Enhancing cotton resistance to Tobacco Mosaic Virus (TMV) [41]
Base Editing	dCas9 or nCas9 + deaminase	sgRNA	ssDNA	PAM: typically 5′-NGG-3′ (SpCas9); varies by Cas9 variant	No DSBs or donor template needed; avoids indels; enables precise C→T or A→G base conversion [42]	Limited editing window and base substitution types (only purine→purine or pyrimidine→pyrimidine) [43,44]	Efficient C→T base editing in rice, wheat, and maize [45]; herbicide tolerance in rice via miRNA target site editing [46]
Prime Editing	nCas9 + M-MLV RT	pegRNA	ssDNA within R-loop generated by pegRNA-nCas9 complex	PAM: typically 5′-NGG-3′ (SpCas9); alternative PAMs available with engineered variants	Enables all 12 base substitutions and small indels without DSBs or donor DNA [26,47]	Editing efficiency is relatively low and context-dependent; pegRNA design and MMR activity influence outcomes [26,48]	Herbicide resistance in rice via saturation mutagenesis of *OsACC1* using prime-editing libraries [49]
Reverse Prime Editing	nCas9-D10A–M-MLV RT ± Rep-X helicase	rpegRNA/pegRNA	Chromosomal DNA (upstream of nick site)	PAM: typically 5′-NGG-3′ (SpCas9); alternative PAMs available with engineered variants	Improves efficiency and fidelity; reduces DSBs and indels [50,51]	Still in early development; editing window design is complex; broad applicability yet to be validated	Expanding editing scope in human cells through reverse prime editing at protein-coding loci such as *BRCA1* and *RPE65* [50]
dCas9 system	dCas9	sgRNA	dsDNA	PAM: typically 5′-NGG-3′ (SpCas9); alternative PAMs available with engineered variants	Lacks nuclease activity; genome remains intact; compatible with CRISPRa/i and live-cell imaging via fusion proteins [52,53]	Complex off-target effects may interfere with screening accuracy [54]	Induction of haploid formation in sweet potato via *IbBBM* activation using dCas9-based activation system [55]

Abbreviations: *TaGW2*—*Triticum aestivum* Grain Weight 2, a negative regulator of grain size and weight in wheat; *OsDL*—*Oryza sativa* Drooping Leaf, involved in vascular development and leaf morphology; *OsALS*—*Oryza sativa* Acetolactate Synthase, encodes a herbicide resistance–associated enzyme in branched-chain amino acid biosynthesis; *OsEPFL9*—*Oryza sativa* Epidermal Patterning Factor-Like 9, encodes a signaling peptide that regulates panicle architecture and grain number via receptor–MAPK pathways; *OsGS3*—*Oryza sativa* Grain Size 3, encodes a G protein–related negative regulator of grain length, widely used in grain yield improvement; *OsACC1*—*Oryza sativa* Acetyl-CoA Carboxylase 1, a herbicide tolerance-related gene; *IbBBM*—*Ipomoea batatas* BABY BOOM, an AP2/ERF transcription factor involved in somatic embryogenesis and haploid induction. PFS refers to the protospacer flanking site, a Cas13-specific recognition motif typically requiring a non-G nucleotide at the 3′ end of the target sequence. Rep-X helicase is an accessory enzyme that facilitates DNA unwinding to enhance the efficiency and fidelity of reverse prime editing.

**Table 3:** Representative genes regulating soybean plant architecture and yield potential.

Gene	Functional Validation	Key Phenotypes	Citation
*Dt1*	Natural variation	Controls stem growth habit; delays vegetative-to-reproductive phase transition	Liu et al. [135]
*Dt2*	Natural variation	Modulates branch number	Liang et al. [127]
*DW1*	EMS-induced mutation	Dwarf phenotype; shortened internodes	Li et al. [118]
*E1*	CRISPR/Cas9 knockout	Reduces photoperiod sensitivity; determinate stem; fewer branches	Wan et al. [136]
*E4*	CRISPR/Cas9 knockout	Promotes early maturation; reduces height and node number	Wu et al. [137]
*GmCRY1s*	CRISPR/Cas9 knockout; Overexpression	Represses stem elongation; enhances lodging resistance and branching	Lyu et al. [120]
*GmDWF1*	CRISPR/Cas9 knockout	Dwarf plants with stable node number; more pods in field trials	Xiang et al. [125]
*GmGA2OX8*	Overexpression; Copy number variation	Reduces shoot length and trailing growth	Wang et al. [119]
*GmILPA1*	Natural mutant (UV-B responsive)	Reduced height under UV-B; shorter internodes and petioles	Sun et al. [126]
*GmJAG1*	CRISPR/Cas9 knockout	Narrower leaves; increased 3- and 4-seeded pods; yield improvement	Cai et al. [133]
*GmLHY*	CRISPR/Cas9 knockout	Reduced height and internode length via GA pathway	Cheng et al. [113]
*GmMRF2*	Overexpression	Earlier flowering in LD; taller plants in both LD and SD	Zhang et al. [138]
*GmNF-YC4*	CRISPR/Cas9 knockout	Early flowering and maturity; adapted to higher latitudes	Cai et al. [139]
*GmPIN1*	CRISPR/Cas9 knockout	Compact architecture; reduced petiole angle; higher yield at density	Zhang et al. [129]
*miR396*	CRISPR/Cas12SF01 knockout	Larger seeds and increased yield in specific regions	Xie et al. [140]
*PH13*	CRISPR/Cas9 knockout; Overexpression	Enhanced shade tolerance and yield under high-density planting	Qin et al. [124]
*RIN1*	CRISPR/Cas9 knockout; γ-ray mutant	Shorter internodes; increased yield at high density; early flowering	Li et al. [123]

Functional validation methods include genome editing, transgenic overexpression, mutagenesis, or exploitation of natural variation. Key phenotypes are reported from cited primary studies. **Abbreviations used in this table:** Gene symbols: *GmTFL1b*—*Glycine max* TERMINAL FLOWER 1b; *GmDWF1*—*Glycine max* DWARF1, involved in brassinosteroid biosynthesis; *GmMRF2*—*Glycine max* MORN-motif Related Factor 2. Technical terms: LD—long day; SD—short day; NSPP—number of seeds per pod; CNV—copy number variation γ-ray mutant—mutant line generated via gamma irradiation; ESE—excessive stem elongation.
